# Kondo peak splitting and Kondo dip induced by a local moment

**DOI:** 10.1038/srep18021

**Published:** 2015-12-10

**Authors:** Pengbin Niu, Yun-Long Shi, Zhu Sun, Yi-Hang Nie, Hong-Gang Luo

**Affiliations:** 1 Institute of Solid State Physics and Department of Physics, Shanxi Datong University, Datong 037009, China; 2 Institute of Theoretical Physics, Shanxi University, Taiyuan 030006, China; 3 Center for Interdisciplinary Studies & Key Laboratory for Magnetism and Magnetic Materials of the MoE, Lanzhou University, Lanzhou 730000, China; 4 Beijing Computational Science Research Center, Beijing 100084, China; 5 Shanxi Provincial Key Laboratory of micro-structural electromagnetic functional materials, Datong 037009, China

## Abstract

Many features like spin-orbit coupling, bias and magnetic fields applied, and so on, can strongly influence the Kondo effect. One of the consequences is Kondo peak splitting. However, Kondo peak splitting led by a local moment has not been investigated systematically. In this research we study theoretically electronic transport through a single-level quantum dot exchange coupled to a local magnetic moment in the Kondo regime. We focus on the Kondo peak splitting induced by an *anisotropic* exchange coupling between the quantum dot and the local moment, which shows *rich* splitting behavior. We consider the cases of a local moment with *S* = 1/2 and *S* = 1. The longitudinal (*z*-component) coupling plays a role of multivalued magnetic fields and the transverse (*x*, *y*-components) coupling lifts the degeneracy of the quantum dot, both of which account for the fine Kondo peak splitting structures. The inter-level or intra-level transition processes are identified in detail. Moreover, we find a Kondo dip at the Fermi level under the proper parameters. The possible experimental observations of these theoretical results should deepen our understanding of Kondo physics.

The Kondo effect^1^,^2^,^3^, first discovered experimentally in the 1930’s as an anomalous increase in the resistance of metals containing magnetic impurities, is a many-body phenomenon. In an electronic transport setup, the Kondo effect can also be realized in quantum dots strongly coupled to external leads^4^. Physically, the higher-order virtual tunneling events can effectively flip and hence screen the local spin on the dot, thus the electrons in the leads and on the dot together form a spin-singlet state. This macroscopically correlated state produces a narrow peak in the dot’s density of states (DOS) around the Fermi level and is known as the spin Kondo effect.

Some recent progress has focused on Kondo peak splitting, which may deepen and enrich our understanding of Kondo effects. Kondo peak splittings may be induced by external factors^5^,^6^,^7^,^8^. In particular, external bias and magnetic field may lead to the Kondo peak splitting^5^. The zero-bias peak splits with external bias voltage, since Kondo resonance is pinned to the Fermi level of each lead. A magnetic field splits the one Kondo peak by the Zeeman effect because that dot’s spin-degenerate energy levels split into separate spin-opposite levels. Moreover, when external ferromagnetic leads are involved, a parallel alignment splits the zero-bias anomaly even in the absence of an external magnetic field, and an antiparallel spin alignment splits Kondo peaks only in the presence of a magnetic field^6^,^7^. Interestingly enough, the valley degree of freedom of external leads reveals a zero-Zeeman-field valley splitting and shows strong temperature dependence, as recently observed in experiments^8^.

Naturally, Kondo peak splittings may also appear when an additional degree of freedom appears^9^,^10^,^11^,^12^,^13^,^14^,^15^,^16^,^17^,^18^,^19^,^20^,^21^,^22^,^23^,^24^,^25^,^26^, such as an orbital degree of freedom (the orbital Kondo effect)^9^ or another spin degree of freedom. In carbon nanotube quantum dots with spin-orbit coupling^10^, splittings emerge when the spin and orbital degrees of freedom couple each other, lift the fourfold degeneracy of a single electron in the dot, and break the SU(4) symmetry^11^. When another spin degree of freedom is involved, such as the large spin of single molecular magnets, a local spin-half impurity, or a magnetic cluster^12^,^13^,^14^,^15^,^16^,^17^,^18^,^19^,^20^,^21^,^22^,^23^,^24^,^25^,^26^, Kondo peak splittings could occur. In single molecular magnets, the magnetic anisotropy of local large spin acts like a magnetic field^24^. In a quantum dot with spin-half impurity, a three-peak Kondo structure characterizes the isotropic exchange interaction between the dot electron and impurity^25^. Finally, in magnetic cluster experiments, a Kondo peak on a Co adatom can be split when the atom interacts with a magnetic Fe cluster. This splitting, depending on the adsorption site, is demonstrated by reversible switching of an adatom between two positions^26^.

We consider theoretically the Kondo peak splitting phenomenon in electronic transport through a single level quantum dot exchange coupled to a local moment, mainly focusing on an anisotropic coupling. Despite the fact that the relevant aspects of the Kondo effect have been widely studied in many models, for example, the T-shaped double quantum dot or two-impurity Kondo problems^27^,^28^,^29^,^30^,^31^,^32^,^33^, the anisotropic exchange coupling between two spin degrees of freedom and its impact on the Kondo effect have not to be explored in detail. One of the challenges of this study is that we have to deal with two anisotropy couplings: the longitudinal anisotropy coupling 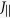 and the transverse anisotropy coupling *J*_⊥_. In the limiting case of strong longitudinal anisotropy coupling, the local moment behaves like a multi-valued quantum magnetic field, and in the isotropic coupling limit, the results show a three-peak Kondo structure consistent with ref. 25. In the general anisotropic case, the degeneracy is lifted and we can identify the entangled states via which Kondo transport occur. The inter-level or intra-level transitions are also identified, which account for the fine Kondo-peak-splitting structures. Moreover, we find a Kondo dip at the Fermi level under proper parameters. To evaluate the dot’s density of states and differential conductance, we use a non-equilibrium Hubbard operator Green’s function method^34^. To calculate the relevant Hubbard operator Green’s functions from the equations of motion, we apply the truncation scheme introduced in ref. 5.

## Results

### Hamiltonian

We consider a model consisting of a single level (SL) quantum dot exchange coupled to a local magnetic moment (spin *S*). This model may be realized in some systems, for instance, magnetic adatoms coupled to a magnetic cluster, quantum dots coupled to a localized magnetic impurity, or a CdTe quantum dot doped with a single metal ion^35^,^36^,^37^,^38^,^39^,^40^,^41^. The exchange coupling is anisotropic and the total Hamiltonian of this model consists of three terms, *H* = *H*_*Leads*_ + *H*_*D*_ + *H*_*T*_, in which


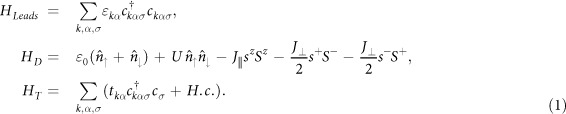


*H*_*Leads*_ is the Hamiltonian of the electrodes, where *ε*_*kα*_ is the energy for free electrons, with *α* = *L*, *R* enumerating left and right leads, and 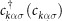 are electronic creation (annihilation) operators in the electrodes, with *σ* = ↑, ↓ representing the up and down electron spins. *H*_*D*_ is the Hamiltonian of the dot, where *ε*_0_ is the SL energy of the dot and *U* is the Coulomb interaction strength, with 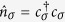 the number operator. 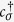 and *c*_*σ*_ are electronic creation and annihilation operators on the SL in the dot. We consider in the calculation below the limit of infinite Coulomb interaction (*U* → ∞), thus only one electron is allowed to occupy the SL. *S*^*z*^ is the *z*-component of the local spin and *s*^*z*^ is the spin of the electron in the SL. The corresponding operators *S*^±^(*s*^±^) are the ladder operators. 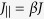 is the *z*-axis exchange coupling between the local spin and SL, and *J*_⊥_ = *αJ* is the *x*-*y* plane exchange coupling, where *α* is the transverse anisotropy parameter and *β* the longitudinal anisotropy parameter. We consider ferromagnetic coupling (*J* > 0) and *α*, *β* ∈ [0, 1]. We expect that the transverse and longitudinal terms in Eq. (1) has different splitting effects on Kondo transport when they appear separately, while they have a combined effect when both terms appear. Thus, there are three limiting cases in our model: i) *β* = 1 and *α* = 0, the strong longitudinal anisotropy coupling case; ii) *β* = 0 and *α* = 1, the strong transverse anisotropy coupling case; and iii) *β* = 1 and *α* = 1, the isotropic coupling case. *H*_*T*_ is the tunnel Hamiltonian between the leads and dot, where *t*_*kα*_ are the tunneling amplitudes. The Hamiltonian *H*_*D*_ is easily diagonalized. In the results and discussions, we will restrict our calculations to the cases of local spin with *S* = 1/2 and *S* = 1, which are the smallest non-trivial values. For *S* = 1/2, two electrons entangle each other, but with an anisotropic exchange coupling. Thus it is worthwhile to provide explicitly the dot’s eigenstates and eigenvalues. The eigenstates and eigenvalues are easily obtained. 
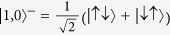
 is a spin triplet state, with energy 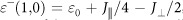, and 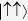 and 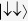 are the other two triplet states, with energy 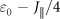. The degeneracy of these triplet states will be lifted in the general anisotropic coupling case. 
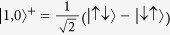
 is the spin singlet state with energy 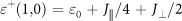.

### Splitting behaviors for *S* = 1/2

**Figure 1:**
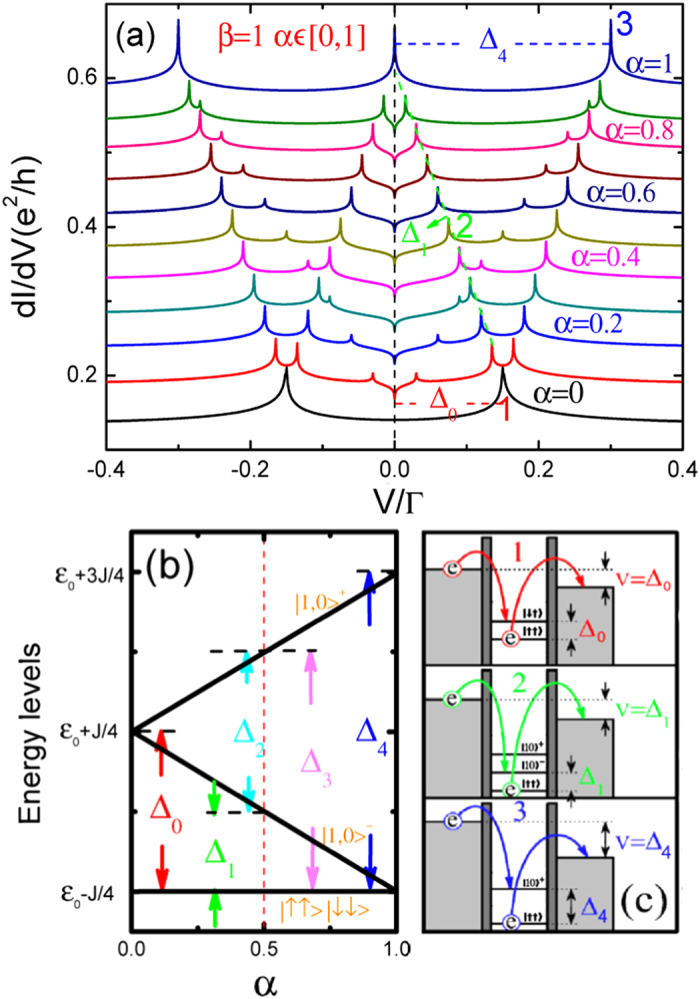
(**a**) *dI*/*dV* versus *V* with fixed longitudinal parameter *β* = 1 and gradual transverse anisotropy parameter, from *α* = 0 to *α* = 1, with *δα* = 0.1 from the bottom up for each curve. The curves for *α* ≠ 0 are shifted upward for clarity. The parameters used are *S* = 1/2, *ε*_0_ = −3, *J* = 0.3, *T* = 0.0001, and the high-energy cutoff *W* = 1000. Δ_0_(red), Δ_1_(green) and Δ_4_(blue) are level spacings corresponding to peaks marked by the numbers 1, 2, and 3. The energy unit is taken as Γ = Γ_*L*_ + Γ_*R*_ = 1. (**b**) The energy spectrum of *H*_*D*_ as a function of the transverse anisotropy parameter *α*. Δ_*i*_ (*i* = 0, 1, 2, 3, 4) are inter-level energy differences. (**c**) Schematic representation of three transition processes producing the peaks marked 1, 2, and 3 in (**a**).

We now present the Kondo peak splitting effects induced by the longitudinal and transverse anisotropy couplings. Experimentally, the low-temperature differential conductance *dI*/*dV* near zero source-drain bias directly reflects the Kondo features in the dot density of states 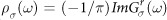. Within the Keldysh formalism^42^,^43^, the transport current is 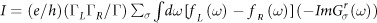
In the numerical results presented below, we assume symmetric dot-lead couplings Γ_*L*_ = Γ_*R*_ and a symmetrically applied source-drain bias (*μ*_*L*_ = *V*/2, *μ*_*R*_ = −*V*/2). The energy unit is taken as Γ = Γ_*L*_ + Γ_*R*_ = 1. Since the presence of the Kondo resonance and its possible splitting play an important role in the transport, it is helpful to first estimate the Kondo temperature *T*_*K*_, which can be calculated from the half width at half maximum of the Kondo resonance^44^,^45^,^46^. For the parameters used in Fig. 1, one gets *T*_*K*_/Γ ≈ 0.026.

**Figure 2:**
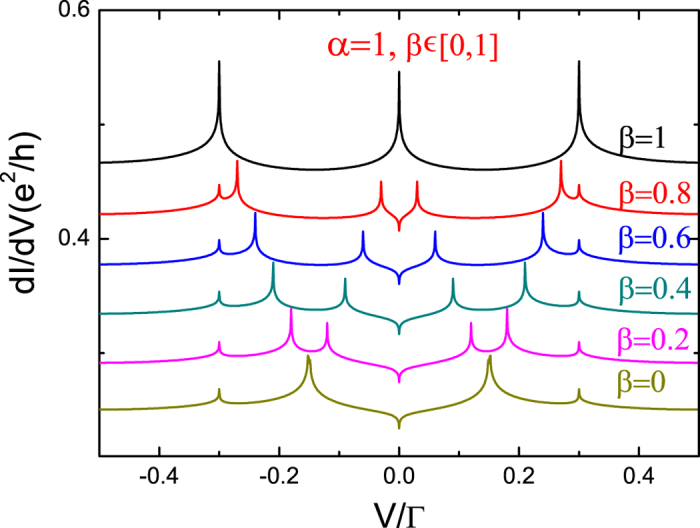
Differential conductance *dI*/*dV* with fixed transverse anisotropy parameter *α* = 1 and gradual longitudinal parameter, from *β* = 0 to *β* = 1, as a function of bias voltage. The curves for *β* ≠ 0 are shifted upward for clarity, the same as in Fig. 1. The parameters are chosen as *S* = 1/2, *ε*_0_ = −3, *J* = 0.3, *T* = 0.0001, and *W* = 1000. Energy is measured in Γ.

Figures 1 and 2 show the splitting effects of the anisotropy parameters *α* and *β* for the minimal non-trivial spin *S* = 1/2. Figure 1 shows the differential conductance, related energy spectrum, and three transition processes. Figure 1(a) shows the differential conductance with a gradual transverse anisotropy parameter, from *α* = 0 to *α* = 1, and fixed longitudinal parameter *β* = 1, in which we can clearly observe the Kondo peak splitting effects. In Fig. 1(b), we present the energy spectrum of *H*_*D*_ and the corresponding eigenstates, which have been calculated in section II. Accordingly, we also get five energy level differences, Δ_0_ = ±*J*/2, 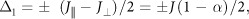 Δ_2_ = ±*J*_⊥_ = ±*αJ*, 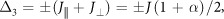, and Δ_4_ = ±*J*, which are shown in Fig. 1(b).

Without the local moment (*J* = 0), the Kondo singlet state transporting through the SL quantum dot manifests itself as a well-known single sharp peak at *V* = 0 in conductance. For the strong longitudinal anisotropy case (*β* = 1 and *α* = 0), the effect of the local moment is equivalent to a magnetic field. The dot’s single level 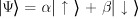 is now coupled with a “ Zeeman field” *S*^*z*^ and split into two energy levels 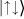 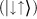 and 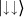 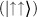, as seen in Fig. 1(b), at *α* = 0. This mechanism is similar to magnetic field induced Kondo peak splitting^5^. The Kondo singlet state tunneling through the dot is a virtual spin-flip process, and thus only inter-level transitions are allowed, which explains the two peaks with *α* = 0 in Fig. 1(a) and why there is no peak at the Fermi surface (*V* = 0). However, the magnetic field here is a “quantum” one, and it can take two values with *ħ*/2 and −*ħ*/2, which are degenerate.

When we turn on the transverse anisotropy parameter *α*, i.e., the term (*J*_⊥_/2)*s*^+^*S*^−^ + (*J*_⊥_/2)*s*^−^*S*^+^, the degeneracy is lifted. In fact, there are two effects of (*J*_⊥_/2)*s*^+^*S*^−^ + (*J*_⊥_/2)*s*^−^*S*^+^: it i) entangles the states 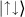 and 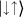 and ii) lifts the degeneracy of 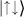 and 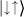 and splits the dot’s energy level. For 0 < *α* < 1, there are seven peaks observed in Fig. 1(a). This splitting, originating from the dot’s energy level splitting, can be explained with energy level differences. When Kondo transport occurs, it can happen via inter-level transitions or intra-level transitions [see schematic representation of three transitions in Fig. 1(c)]. Δ_*i*_(*i* = 1, 2, 3) correspond to six inter-level transitions, and the peak at *V* = 0 corresponds to intra-level transitions. Thus, there are seven peaks observed. Specifically, when *α* ≠ 0, three separated states form: 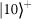, 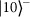, and the fully polarized state 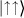 or 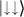. Kondo transport occurs via either one or two of these states. For example, the intra-level transition 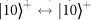 or 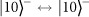 induce the Kondo peak at *V* = 0 and the inter-level transitions 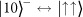 induce the Kondo peaks at *V* = ±Δ_1_. It seems that the position of the peak at *V* = 0 is the same as for a single-level quantum dot, but the underlying mechanism is different. The former is a two-electron entangled state, while the latter is a one-electron state.

When *α* = 1, the exchange coupling becomes isotropic. This isotropic interaction induces a three-peak structure. This structure is also observed in spin-orbit coupling^11^, which can be formulated as 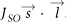. When the anisotropic interaction develops into an isotropic one, one observes that the number of Kondo peaks is reduced. This is because the entangled state 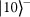 becomes degenerate with the fully polarized states [see Fig. 1(b)].

We now focus on the strong transverse anisotropy coupling case (*β* = 0 and *α* = 1), which is shown in Fig. 2. In Fig. 2 we present the results with a gradual longitudinal anisotropy parameter, from *β* = 0 to *β* = 1, with fixed transverse parameter *α* = 1. For 0 < *β* < 1, there are also seven peaks observed, which confirm the result in Fig. 1(a). For *β* = 0, we observe five Kondo peaks, which are located at *V* = 0, ±*J*_⊥_/2 and ±*J*_⊥_. These five peaks may also be explained by using the language of intra- and inter-level transitions.

### Splitting behaviors for *S* = 1

Up to now, we have studied the conductance diagram of the system as a function of the anisotropy parameters for *S* = 1/2. However, for a given magnetic cluster or molecule, the local spin usually has the value *S* > 1/2. Here, we restrict our discussion to the case of a spin *S* = 1. We will study the behavior of the conductance varying the anisotropy parameters. The goal is to understand how the values of the local spin influence the behavior of the conductance and Kondo peak splittings.

**Figure 3:**
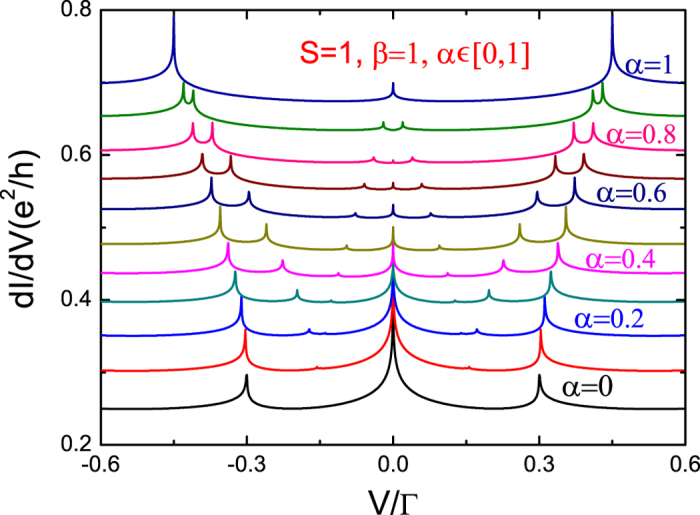
Differential conductance *dI*/*dV* with fixed longitudinal parameter and gradual transverse parameter as a function of bias voltage for *S* = 1. The parameters are chosen as *ε*_0_ = −3, *J* = 0.3, *T* = 0.0001, and *W* = 1000. Energy is measured in Γ.

**Figure 4:**
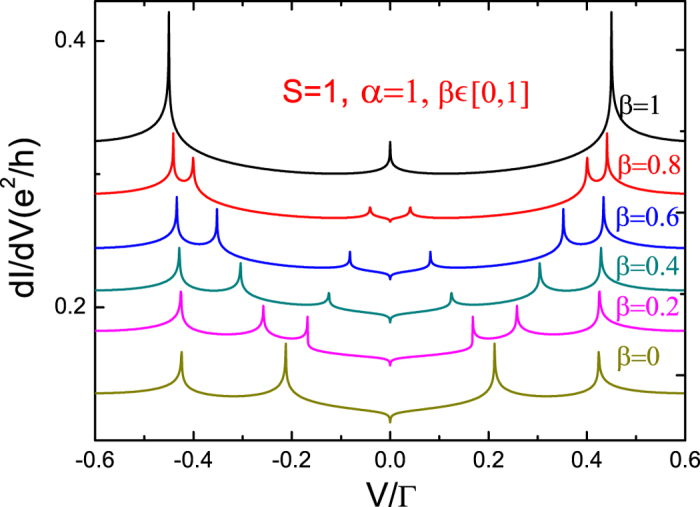
Differential conductance *dI*/*dV* with fixed transverse parameter and gradual longitudinal parameter as a function of bias voltage for *S* = 1. The other parameters are the same as in Fig. 3. Energy is measured in Γ.

Figures 3 and 4 present the differential conductance for integer spin *S* = 1. As with *S* = 1/2, we vary one of the anisotropy parameters while the other one remains fixed. As seen in Fig. 3, when *β* = 1 and 0 < *α* < 1, seven peaks are still observed. This is because the energy levels are split into three as before, and are enumerated as *ε*^+^(1, ±1/2), *ε*^−^(1, ±1/2), and the fully polarized states *ε*(↑, *S*). However, one can deduce that when *S* grows greater, energy levels increase, and likewise with energy level differences and Kondo peaks. When *β* = 1 and *α* = 0, one has the strong longitudinal anisotropy case. In this situation, the quantum magnetic field *S*^*z*^ takes three values, −*ħ*, *ħ*, and 0. As a result, at *V* = ±*J* there are two peaks corresponding to inter-level transitions and for *S*^*z*^ = 0 it is equivalent to a zero-Zeeman-field corresponding to the Kondo peak at *V* = 0. For the strong transverse anisotropic case (*β* = 0 and *α* = 1), we observe five peaks, which are located at 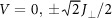 and 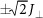, as seen in Fig. 4. In Figs 1, 2, 3, 4, one can observe a Kondo dip at the Fermi surface (*V* = 0). This dip is a feature of anisotropic coupling. Under proper parameters, the anisotropic coupling will change the sign of Kondo self-energy at *V* = 0 and induce the Kondo dip. Moreover, one can conclude that the interplay of 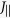, *J*_⊥_, and temperature can strongly influence the Kondo resonance at the Fermi surface, i.e., the formation or destruction of a Kondo peak (dip) at Fermi surface.

## Methods

The non-equilibrium Green’s function method has been widely used in the discussion of Kondo effects in quantum dots^5^,^47^–5^1^. In order to handle the large spin involved in many problems, we use the Hubbard operator Green’s function method^34^, which has been used to solve the transport problems in the linear response, non-linear response, and Kondo regimes^52^,^53^,^54^. In this Hubbard operator representation, the electron operators in the dot are rewritten as 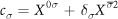 with *δ*_*σ*_ = +1(−1) for *σ* = ↑(↓), 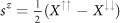, *s*^+^ = *X*^↑↓^ and *s*^−^ = *X*^↓↑^. In the limit of infinite Coulomb interaction (*U* → ∞), double-occupation is forbidden and *c*_*σ*_ = *X*^0*σ*^. The large spin operators can be expressed as^34^ 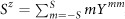, 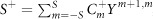, and 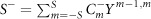, with *S* representing the large spin quantum number and 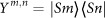. Hence, the total Hamilton is rewritten as


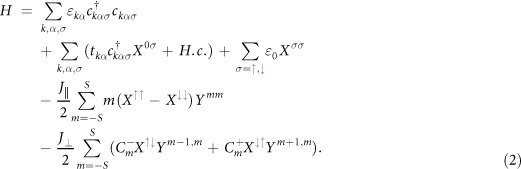


The retarded Green’s function 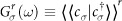, which will be needed in the density of states 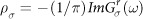 and transport calculations, is expressed as


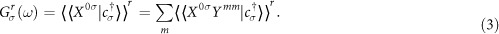


We use the equation of motion method to calculate the retarded Green’s function. Although the equation of motion method is an approximate one due to truncation scheme, it was shown that this method can capture qualitatively the Kondo physics^48^. The equation of motion of 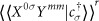 reads


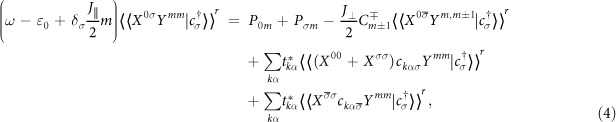


where the upper (lower) sign applies for *σ* = ↑(↓). The averages are defined as *P*_0*m*_ ≡ 〈*X*^00^*Y*^*mm*^〉, *P*_*σm*_ ≡ 〈*X*^*σσ*^*Y*^*mm*^〉. One may notice that in the above equation there is a newly generated Green’s function 
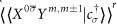
 that has the same order as 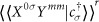. Other newly generated Green’s functions are high-order ones that contain just one lead-operator. The equation of motion of these high-order Green’s functions will generate higher-order ones containing two lead-operators. Here is an example:


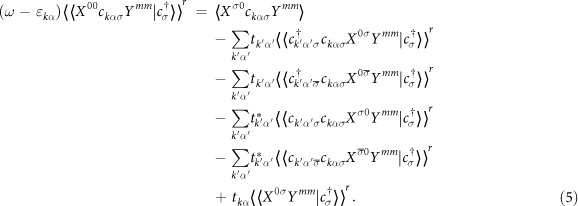


We truncate these higher-order Green’s functions following the procedure proposed by Meir, Wingreen, and Lee^5^, in which we pull the operator pair of leads out of the Green’s functions and regard them as an average. This truncation approximation captures Kondo physics well when the temperature of the system is low.

After this truncation, Eq. (5) becomes


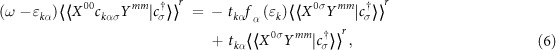


in which the average 〈*X*^*σ*0^*c*_*kασ*_*Y*^*mm*^〉 is simply discarded, 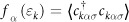 is the Fermi function, and other averages of lead operator pairs equal zero.

In the same way, one can obtain the equation of motion of 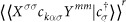, which couples with the other three high-order Green’s functions 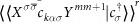 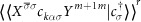 and 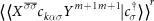. In the calculation procedure, one may notice that the high-order Green’s functions containing one operator *c*_*kασ*_ couple each other, while the high-order Green’s functions with 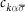 couple each other. These are 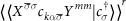, 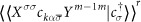, 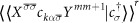, and 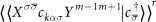. After solving these simultaneous equations, we get the solutions of these one-lead-operator Green’s functions. Inserting them back into Eq. (4) and the equation of motion of 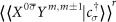, we get the analytical results of the retarded dot Green’s function


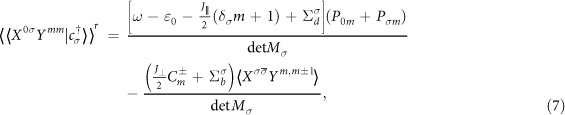


where the matrix *M*_*σ*_ is defined as


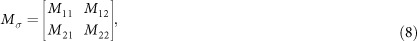


with 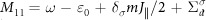, 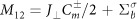, 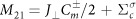, and 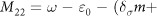

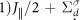
.

This analytical expression for the retarded Green’s function is one of the main results in this work, and it is the starting point for further numerical discussions. In Eq. (7), 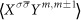 is an average appearing in the equation of motion of 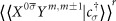. One may notice that this average has a self-adjoint average, 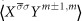. In Green’s function theory, the averages can be related with Green’s functions via the fluctuation-dissipation theorem 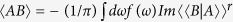, in which *f*(*ω*) is the Fermi function. If considering the non-equilibrium situation, one can simply impose bias onto the Fermi function of this theorem, i.e., 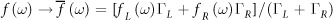. In order to relate the averages (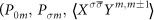 and 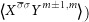 with Green’s functions, one may find that the Green’s functions 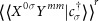, 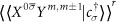, 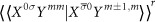, and 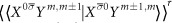 are useful. In Eqs. (7) and (8) 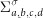 are combinations of Kondo self-energies that characterize the low-temperature Kondo transport properties of the dot. The Kondo self-energies are given as follows


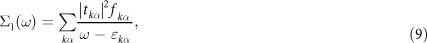



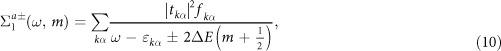



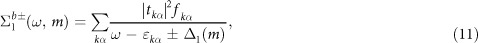



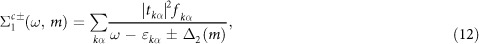


where Δ_1,2_ are defined as


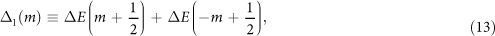



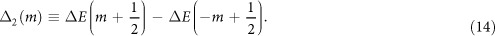


In principle, in Eqs. (9)–(12), 86, 87, 88 at the pole positions of the self-energies, the Kondo peaks should manifest themselves in density of states and conductance. However, at the same time, these self-energies are modulated by parameters of the system. For example, 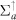 is a combination of Kondo self-energies:


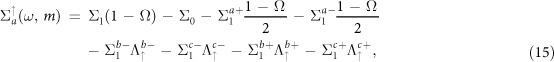


where the coefficients Ω(*m*) and 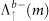 (as examples) are defined as


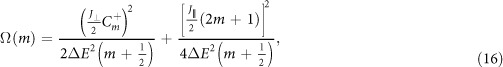


and


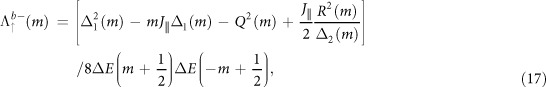


where 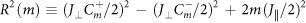 and 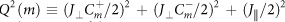 are defined for conciseness. These coefficients are dimensionless combinations of the anisotropy parameters 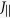 and *J*_⊥_.

## Additional Information

**How to cite this article**: Niu, P. *et al*. Kondo peak splitting and Kondo dip induced by a local moment. *Sci. Rep.* **5**, 18021; doi: 10.1038/srep18021 (2015).
